# HIV policy implementation in two health and demographic surveillance sites in Uganda: findings from a national policy review, health facility surveys and key informant interviews

**DOI:** 10.1186/s13012-017-0574-z

**Published:** 2017-04-05

**Authors:** Ellen McRobie, Alison Wringe, Jessica Nakiyingi-Miiro, Francis Kiweewa, Tom Lutalo, Gertrude Nakigozi, Jim Todd, Jeffrey William Eaton, Basia Zaba, Kathryn Church

**Affiliations:** 1 Imperial College London, Norfolk Place, London, W2 1PG UK; 2 London School of Hygiene and Tropical Medicine, Keppel Street, London, UK; 3 MRC/UVRI Uganda Research Institute on AIDS, Entebbe, Uganda; 4 Makerere University Walter Reed Project, Kampala, Uganda; 5 Rakai Health Sciences Program, Entebbe, Uganda

**Keywords:** HIV, HIV policies, Health services, Uganda

## Abstract

**Background:**

Successful HIV testing, care and treatment policy implementation is essential for realising the reductions in morbidity and mortality those policies are designed to target. While adoption of new HIV policies is rapid, less is known about the facility-level implementation of new policies and the factors influencing this.

**Methods:**

We assessed implementation of national policies about HIV testing, treatment and retention at health facilities serving two health and demographic surveillance sites (HDSS) (10 in Kyamulibwa, 14 in Rakai). Ugandan Ministry of Health HIV policy documents were reviewed in 2013, and pre-determined indicators were extracted relating to the content and nature of guidance on HIV service provision. Facility-level policy implementation was assessed via a structured questionnaire administered to in-charge staff from each health facility. Implementation of policies was classified as wide (≥75% facilities), partial (26–74% facilities) or minimal (≤25% facilities). Semi-structured interviews were conducted with key informants (policy-makers, implementers, researchers) to identify factors influencing implementation; data were analysed using the Framework Method of thematic analysis.

**Results:**

Most policies were widely implemented in both HDSS (free testing, free antiretroviral treatment (ART), WHO first-line regimen as standard, Option B+). Both had notable implementation gaps for policies relating to retention on treatment (availability of nutritional supplements, support groups or isoniazid preventive therapy). Rakai implemented more policies relating to provision of antiretroviral treatment than Kyamulibwa and performed better on quality of care indicators, such as frequency of stock-outs. Factors facilitating implementation were donor investment and support, strong scientific evidence, low policy complexity, phased implementation and effective planning. Limited human resources, infrastructure and health management information systems were perceived as major barriers to effective implementation.

**Conclusions:**

Most HIV policies were widely implemented in the two settings; however, gaps in implementation coverage prevail and the value of ensuring complete coverage of existing policies should be considered against the adoption of new policies in regard to resource needs and health benefits.

## Background

The response to the HIV/AIDS epidemic has been unprecedented in its accumulation and application of research knowledge for policy and practice development [1]. Significant progress has been made, as marked by the achievement of Millennium Development Goal 6, with an estimated 30 million new HIV infections averted and 7.8 million AIDS-related deaths prevented since 2000 [2]. The effectiveness of treatment programmes in reducing HIV-related mortality, however, is predicated on effective implementation of policies around HIV testing, care and treatment to ensure timely treatment initiation and retention [3]. Recent systematic reviews suggest heavy attrition of people living with HIV (PLHIV) at all stages of the care continuum, resulting in persistently higher mortality in the HIV-positive population compared to those who are HIV negative [4]. This raises the question about the extent to which appropriate policies exist, the extent to which they are implemented and factors that facilitate or disable policy implementation.

Uganda has a large and growing population of PLHIV (currently estimated 1.6 million) [5]. Data from two health and demographic surveillance sites (HDSS) in rural Uganda indicate that while mortality had substantially declined since the introduction of anti-retroviral treatment (ART) in 2004 (from 90 deaths per 1000 person years in Kyamulibwa and 96.2 deaths per 1000 person years in Rakai to 22.7 deaths and 22.5 deaths per 1000 person years, respectively), there continues to be substantially higher mortality in the HIV-positive population compared to the HIV-negative population (HIV-negative mortality rates were 8.6 deaths and 3.4 deaths per 1000 person years for Kyamulibwa and Rakai, respectively) (Table 1) [6]. Reviewing current policy in relation to its implementation in health facilities to identify gaps in service provision in these two settings may provide insight into the factors contributing to the higher mortality rates observed in PLHIV.

**Table 1:** Background characteristics of the study sites

	Kyamulibwa	Rakai
Site history		
Start of demographic data collection and serosurveys	Nov-89	Apr-99
Number of clinics (surveyed/total in HDSS)	10/3^a^	14/17
Size (km^2^)	28	320
Adults (15+ years) under surveillance, 2013	9697	20,055
ART indicators		
Introduction of ART	Jan-04	Jun-04
Full ART implementation	Jan-05	Jun-06
HIV indicators		
HIV prevalence (2013) (%)	9.4	12.5
HIV−ve mortality rate, 2009–2011 (per 1000)	8.6	3.4
Pre-ART HIV+ve mortality rate (2000–2003) (per 1000)	90	96.2
Post-ART HIV+ve mortality rate (2009–2011) (per 1000)	22.7	22.5

Source: [6, 22]

^a^Six facilities outside of the HDSS that also serve the population of Kyamulibwa were also included in the analysis

**Fig. 1:**
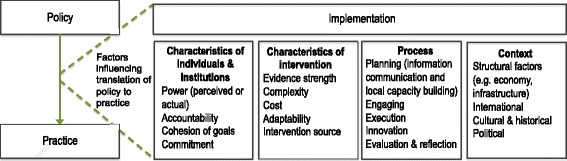
Theoretical framework to guide the analysis of factors influencing the translation of policy to practice

A study by Church et al. on behalf of the network for Analysing Longitudinal Population-based HIV data in Africa (ALPHA) reviewed the content of policies relating to HIV testing, treatment and retention in six sub-Saharan African countries (Kenya, Malawi, South Africa, Tanzania, Uganda, Zimbabwe) and found substantial variability in national policy adoption and the extent to which policies adhere to WHO guidelines [7]. While policy reviews are valuable in terms of understanding national-level policy formulation, their translation into practice can often occur incompletely or with unexpected outcomes, and these resulting gaps in care provision may be hypothesised to explain observed health outcomes. The extent to which policies are implemented in different settings is unknown, and there may be variation by facility level or at specific stages on the continuum of HIV care. Political, social or structural factors are likely to play a role in differences between countries about how policy is put into practice [8, 9]. Figure 1 presents a framework of factors that are considered to be important in the health policy implementation process. The framework combines theoretical insights from Walt and Gilson’s health policy triangle (actors, content, context, process), as well as Damschroder’s Consolidated Framework on Implementation Research (CFIR) [10, 11]. This latter framework incorporates five domains (characteristics of the intervention, characteristics of individuals, process, inner and outer setting) and 12 constructs within those domains. The adapted framework is used here to understand political, social and structural processes influencing HIV policy implementation.

Reviewing policy implementation status, and assessing the influences that contribute, is critical for ensuring a more effective policy cycle, from formulation through to delivery, and back into re-formulation. This study aims to compare the status of implementation of policies promoting access to HIV testing, treatment and retention on treatment for facilities serving the population of two health and demographic surveillance sites (HDSS) in southern Uganda: Kyamulibwa and Rakai.

## Methods

### Study setting

This analysis forms part of a larger study by ALPHA on HIV-related mortality; thus, the two Ugandan HDSS sites that contribute data to the ALPHA Network are investigated. Both HDSS are located in pre-dominantly rural Ugandan regions. Kyamulibwa, run by the UK Medical Research Council/Uganda Virus Research Institute, is located within Kalungu District and began collecting demographic and health data on the population in 1989 [12]. Rakai, run by the Rakai Health Sciences Program, is located within Rakai District and began its open-community cohort for 15–49 year olds in 1994 [12]. Background characteristics of the two sites are displayed in Table 1.

### Policy review and document analysis

A review of Ugandan HIV policy documents was conducted in the study by Church et al. [5]. This involved an online and library search for any relevant ministerial or national guideline documents on HIV counselling and testing (HCT), prevention of mother to child transmission (PMTCT) and HIV care and treatment published between 2003 and 2013. Staff from the Ministry of Health were also contacted in person in order to access all iterations of guidelines. The review captured detailed policy information (including year of formulation and any subsequent changes) for 54 indicators influencing access to HCT, access to care and treatment and retention in care. The content was reviewed and compared against successive iterations of WHO guidelines.

### Health facility survey data and analysis

A health facility survey was subsequently carried out in 2013 by the ALPHA network to investigate reported practice in areas considered to influence access to HIV care services [13]. Detailed methods are described elsewhere [13]. In total, 24 facilities were surveyed. In Kyamulibwa, this included the three facilities located within the HDSS and a further six on the edge of or just outside the HDSS used by residents (*n* = 10). In Rakai, all facilities were surveyed except three very small clinics (*n* = 14). A structured questionnaire was administered to in-charge staff within three types of service units of participating facilities: HCT, PMTCT and ART services (the latter two where operational). Descriptive statistics were produced using STATA 13.0, including cross-tabulations of key indicators by site and by size of facility. Statistical tests were not conducted, as these were non-random samples, and not necessarily representative of a wider population of health facilities. Where data were missing, the denominator reflects the proportion of responses only, highlighted in the table.

### Analysis to assess facility-level policy implementation

The policy review classified policies as explicit or not explicit (absent, vague or imprecise). Implementation of each policy was assessed using data from the health facility survey. Policies were defined as widely implemented if equal to or more than 75% facilities enacted the policy, partially implemented if implementation occurred in 26–74% of facilities and minimally implemented if 25% or fewer facilities reported implementation. Results were colour-coded green-orange-red to represent the degree of implementation, and dark or light shading was used to illustrate if policy was explicit or not explicit. Results were grouped into different policy domains, derived from the conceptual framework developed by Church et al.: service access and coverage, coordination of patient care and tracking, support to PLHIV, medical management and quality of care [7]. Findings for quality of care are presented separately.

### Key informant interviews

Seven semi-structured interviews were conducted with key informants who were purposively sampled based on their knowledge of the Ugandan HIV policy and practice context, including individuals with experience of policymaking (Ministry of Health, Uganda AIDS Commission), programme implementation (a donor partner relevant to the HDSS, a district health officer for a HDSS region and senior management from each HDSS) or HIV policy research in Uganda. Respondents were invited to participate by email, and informed consent was obtained. Interviews were conducted after the policy and practice comparisons had been undertaken to enable discussion of site-specific implementation gaps and to explore differences between sites. Interviews were conducted in-person or by telephone, recorded and transcribed. Follow-up interviews were conducted with respondents most familiar with programme implementation in the HDSS to investigate site-specific differences. Names and titles were removed to protect anonymity. Data were managed in Nvivo10. A deductive coding approach was used based on a priori knowledge of implementation theories, frameworks and models. The framework approach was used for analysis: data from each node (code) were extracted and entered into a matrix and memos were elaborated in order to reflect on the data and consider possible interpretations.

## Results

An overview of the facilities surveyed is presented in Table 2. Facilities were large health centres (serving a population of approx. 20,000 with both out- and in- patient services) or smaller out-patient-only clinics (60% in Kyamulibwa, 85.7% in Rakai). More facilities were government-run in Rakai (85.7%) than those in Kyamulibwa (40%). All facilities provided HCT and HIV care and treatment; in Kyamulibwa, only 80% had an ANC clinic with each of these providing PMTCT. Human resources were similar across both sites in regard to median numbers of clinicians, nurses/midwives and counsellors in facilities; however, there were stark disparities in the staff to client ratios: Rakai had much higher numbers of patients per staff member per week for HCT (58 clients per week, compared to 19 per week in Kyamulibwa) and ART (49 per week, compared to 3 per week in Kyamulibwa). Staff turnover was broadly similar, but with a larger range in Kyamulibwa.

**Table 2:** Overview of Kyamulibwa and Rakai HDSS included in facility survey

	Kyamulibwa		Rakai	
Total no. of clinics (*n*(%))	10	(100.0)	14	(100.0)
Type of facility (*n*(%))				
Large clinic/small health center	6	(60.0)	12	(85.7)
Large health center/hospital	4	(40.0)	2	(14.3)
Management authority (*n*(%))				
Government	4	(40.0)	12	(85.7)
Faith-based organisation	3	(30.0)	0	(0.0)
Other NGO	3	(30.0)	2	(14.3)
HIV-related services (*n*(%))				
HIV counselling and testing	10	(100.0)	14	(100.0)
PMTCT	8	(80.0)	14	(100.0)
HIV care (incl. pre-ART) and treatment	10	(100.0)	14	(100.0)
HR and patient load (median (range))				
No. of clinicians^a^	3.0	(2.0–6.5)	2.0	(0.0–7.0)
No. of nurses/midwives	4.0	(0.0–9.0)	3.3	(1.0–8.0)
No. of counsellors	1.5	(0.0–67.0)^d^	1.0	(0.0–10.0)
No. of HIV testing clients/week	19.0	(1–2011.0)^d^	58	(4–262)
No. of ART clients/week	3.0	(0–270.0)	49	(19–508.0)
No. of weekly HIV testing clients/staff^b^	1.8	(0.0–4.3)	6.9	(0.5–29.3)
No. of weekly ART clients/clinician or nurse	0.3	(0.0–29.8)	10.7	(4.8–253.9)
Staff turnover^c^	7.8	(0–20.0)	4.8	(2.0–10.0)

Source [13]

^a^Doctor, clinical officer, and assistant medical officer

^b^Nurse, midwife, nursing aide, counsellor or community outreach worker

^c^Total staff (nurses, clinicians, aides, counsellors, outreach)/number left in past year

^d^One facility included all outreach workers, which represents the high end of the range

### Findings of reported practice compared to policy

Across both sites, the majority of policies relating to HIV testing, treatment and retention in care were widely implemented; however, there was some variability between sites. The year of national policy adoption does not appear to influence implementation.

### Implementation of policies promoting access to HIV testing

Table 3 summarises policy implementation for HCT services. Of 12 explicit policies reviewed, eight were widely implemented in both Kyamulibwa and Rakai (six were the same policies). Implementation gaps differed between sites: fewer facilities in Kyamulibwa implemented policies relating to service coverage and access, and facilities in Rakai implemented fewer policies to support PLHIV. Both sites reported only partial implementation of two explicit polices on patient coordination/tracking: testing repeated 3 months after first test in pregnancy and/or in the third trimester (50% in Kyamulibwa, 35.7% in Rakai), and HIV test result recorded in patient-retained card (50% both sites). Regarding indicators for which there was no explicit policy in this setting: no facilities in Kyamulibwa and one in Rakai provided dedicated testing for most-at-risk populations (MARPs) such as commercial sex workers, injection drug users and men who have sex with men. While policy states that testing should be provided to MARPs, the guidance does not explicitly name these groups, as some are not legally recognised.

**Table 3:**
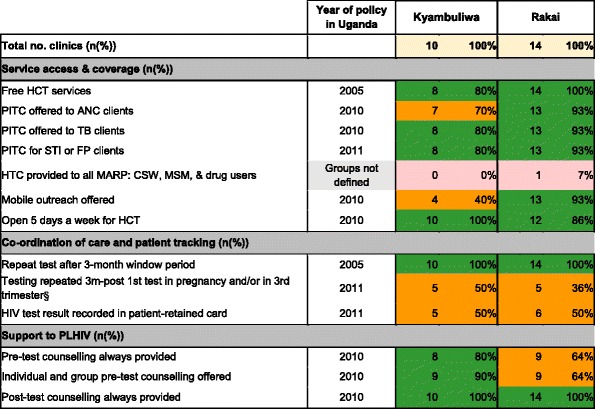

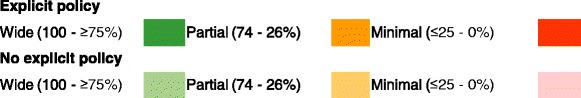
Implementation of policies influencing access to HIV counselling and testing

*ANC* antenatal clinic, *CSW* commercial sex worker, *MSM* men who have sex with men

^§^In clinics with ANC only

### Implementation of policies promoting access to HIV treatment and PMTCT

Table 4 summarises implementation of policies influencing access to HIV treatment (measured in either PMTCT or ART clinics). Out of 17 explicit policies, 15 were widely implemented in Rakai versus 13 in Kyamulibwa; nine of these were the same indicators. Kyamulibwa reported partial implementation for the five remaining explicit policies: free ART services (60% of facilities), ART initiation and provision 5 days a week (30%), ART initiation performed by nurses and midwives (70%) and renal function tests prior to ART initiation (40%). For two explicit policies, there was minimal implementation coverage across facilities in Rakai: renal function tests prior to ART initiation (0%) and two adherence counselling sessions prior to ART (50%). No facilities across Kyamulibwa or Rakai reported offering treatment services to MARP; however, as with HCT for these groups, policy did not name types of MARPs explicitly. One facility in Kyamulibwa reported initiating treatment at 500 CD4 cells/mm^3^, despite this not being policy at the time of the survey.

**Table 4:**
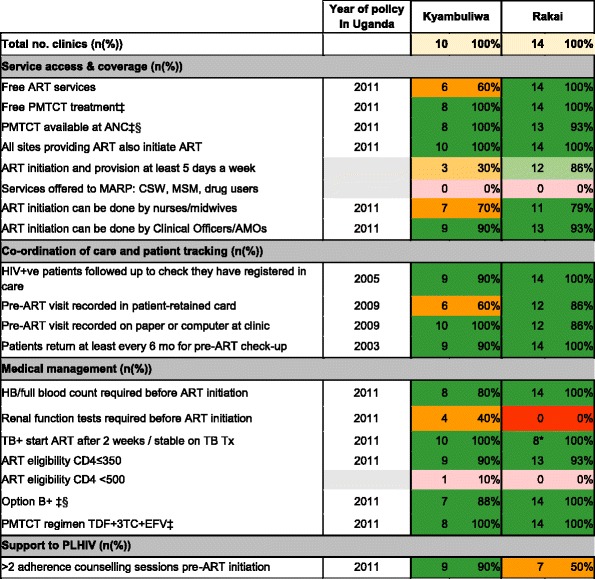

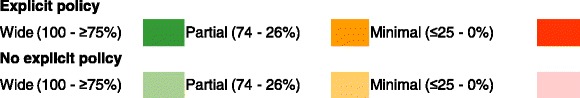
Implementation of policies influencing access to HIV treatment (measured in PMTCT and ART units)

Source: [23–25]

*ANC* antenatal clinic, *CSW* commercial sex worker, *MSM* men who have sex with men, *AMO* accredited medical officer

*>10% sites missing data

^‡^Total number of facilities providing PMTCT in Kyamulibwa is 8

^§^ARV prophylaxis or treatment for mother and prophylaxis for baby

### Implementation of policies influencing retention on ART

Table 5 summarises implementation of policies influencing retention on ART. Of 12 explicit policies, nine were widely implemented in Kyamulibwa compared to eight in Rakai. The sites differed in implementation of explicit policy on TB integration within facilities (100% of facilities in Kyamulibwa versus 69.2% in Rakai). Implementation of policies on support to PLHIV was weaker. A higher proportion of facilities in Rakai implemented support groups (available either onsite or through referral within the district) and home-based care (93% for both), compared to Kyamulibwa (50% and 70%, respectively). There was partial implementation of explicit policy on provision of nutritional supplements to malnourished PLHIV in both Kyamulibwa and Rakai. Only 14.3% facilities in Rakai reported conducting individual counselling over group counselling for adherence, despite policy documents noting the value added in ensuring one-to-one counselling. No facilities in both HDSS offered or had in stock prophylactic isoniazid preventive therapy (IPT). Two facilities in Kyamulibwa reported implementation of home visits following poor adherence despite this not being national policy.

**Table 5:**
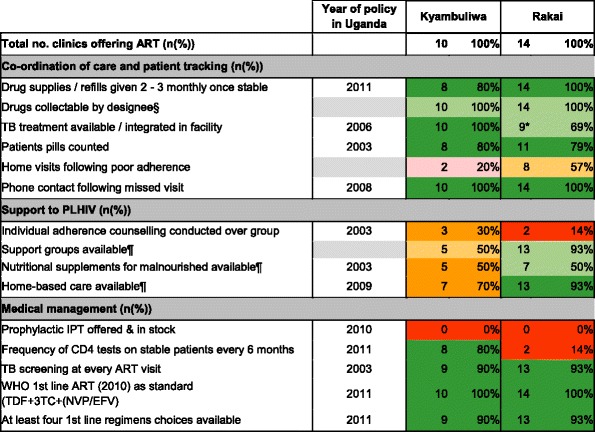

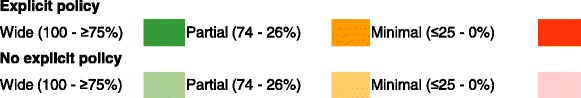
Implementation of policies influencing retention on ART

Source: [23, 24, 26]

*<10% sites missing data

^§^Not more than twice

^¶^Onsite or through referral within district

### Quality of care indicators

Overall, Kyamulibwa performed less well on the quality of care indicators investigated compared to Rakai. The difference in stock-outs of testing kits between sites was particularly stark: only one facility in Rakai reported stock-out, which was also classified as frequent (i.e. lasting >=2 weeks), whereas 70% of facilities in Kyamulibwa reported stock-outs in the past year and 50% reported these as frequent. The same number of facilities in both sites reported stock-outs of ARVs (20% in Kyamulibwa, 14% in Rakai) and treatments for OIs (30% in Kyamulibwa, 21% in Rakai) in the past year. Only half of the facilities in both sites reported having seen national testing guidelines, but more facilities in both sites reported having seen national treatment guidelines (Table 6).

**Table 6:** Facility implementation of quality of care indicators

	Kyamulibwa		Rakai	
Total no. of clinics (*n*(%))	10	(100)	14	(100)
National testing guidelines available^a^	5	(50)	7	(50)
National treatment guidelines available^a^	7	(70)	12	(86)
≤1 staff received HIV testing training in past 2 years	4	(40)	13^a^	(100)
QOC audits at least once/year	10	(100)	11^a^	(85)
At least one test kit stock-out in past year	7	(70)	1	(7)
Frequent test kit stock-outs^b^	5	(50)	1	(7)
CTX prophylaxis in stock in pre-ART	10	(100)	14	(100)
At least one stock-out 1st line ARVs in past year	2	(20)	2	(14)
Frequent stock-out of 1st line ARVs^b^	2	(20)	2	(14)
At least one stock-out of OI drugs in past year^c^	3	(30)	3	(21)
Frequent stock of OI prophylaxis^b^	3	(30)	2	(14)

*QOC* quality of care, *CTX* co-trimoxazole, *OI* opportunistic infection

^a^Seen or not seen, any guideline

^b^More than once in past year or one stock-out lasting for over 2 weeks

^c^Co-trimoxazole, fluconazole or isoniazid preventive therapy (IPT)

### Influences on policy implementation: findings from key informants

Seven informants (four male, three female) with knowledge of the two HDSS were interviewed. Informants shed light on the patterns of reported implementation, possible reasons for differences between sites and the diverse political, social, structural factors influencing the translation of policy into practice in the Ugandan setting. Key informants were largely successful at predicting, when unprompted, which policies were well implemented and where implementation gaps existed in the two HDSS and along the treatment cascade. Reporting of reasons for differences was highly consistent between respondents, but senior members of the HDSS and the District Health Officer were most aware of implementation gaps. Findings have been detailed below under the domains detailed in Fig. 1 (characteristics of individuals and institutions, characteristics of intervention, processes and context).

### Characteristics of individuals and institutions

When queried on the main reason for implementation gaps observed, all respondents noted the necessity of donor financial support to ensure implementation, in particular for treatment services from the United States President’s Emergency Plan for AIDS Relief (PEPFAR) and the Global Fund to Fight AIDS, TB, and Malaria. In Rakai, the adequate provision of ART, in particular, was seen as contingent upon PEPFAR’s funding for medication:

For example, when [the WHO] said they upgrade to the CD4 500 we had a commitment from PEPFAR that they will be able to supply us with drugs for the additional number of people who are now eligible. Unless that is done we can’t support it. [R5]

The widespread implementation of many policies relating to treatment in Rakai was attributed to the volume of funding from PEPFAR, the Bill and Melinda Gates Foundation and support from the Department of Health, funds which were not received to the same extent in Kyamulibwa. Additionally, respondents noted that as facilities in the Rakai HDSS are pre-dominantly government-run and largely have the same funding source, there was coherence in communication among the facilities.

When respondents were asked to name a policy that they were confident would be implemented in facilities, all stated Option B+, the initiation of lifelong treatment to pregnant and breastfeeding women. Several respondents commented how many individuals and institutions from the global level to health workers on the ground had worked together to ensure facility implementation:

…the leadership of Ministry also really was very supportive of PMTCT as a whole… Then WHO supported us through the guidelines, and UNICEF [United Nations International Children’s Emergency Fund]. Everyone, all the organizations were very instrumental. [R3]

Some respondents noted, however, that national priorities were not always aligned with donor priorities stating that it was “partner interest that takes precedent” [R3] and that policy evolution was often “donor driven” [R3]. Most noted that WHO and others pushed policy changes, despite Uganda and, in particular, the Rakai HDSS, being a leading setting for HIV research and policy innovation. Donor prioritisation and investment toward high-impact interventions were seen to result in insufficient attention being paid to public health infrastructure, to the point of a “hollowing out” [R4], as one respondent put it. Donors were considered to focus more on provision of medicines, rather than on traditional prevention activities. Further, recent donor funding declines were attributed to the cessation in provision of services to support PLHIV in HDSS in Kyamulibwa:

Those sorts of soft cuddly things really have been removed… For example, MRC [Medical Research Council] used to have a lot of community helpers groups, but in 2011 after that 5-year funding phase they just said no and that the money from DFID is just not there anymore, so it disappears. [R2]

### Characteristics of the intervention

Many respondents underlined the importance of strong scientific evidence to facilitate policy implementation: “in case there is some ground-breaking evidence, like say PMTCT option B+, implementation will be done earlier” [R1]. Option B+ roll-out was also facilitated by the ability to rapidly view results, with demonstrated impact on newborn HIV incidence in a short time frame providing additional motivation for donor support of this policy:

Option B+ has given us tangible results. Results that are visible by each and everyone[…], it’s one of those that have been implemented so well and we are increasingly getting HIV-negative babies born to HIV-positive mothers. [R6]

The low complexity of the task also enabled Option B+ roll-out. Since it was a simplification of the previous regimen (option A), it required minimal staff training or adjustment to practice and could now be performed by lower cadres of staff (nurses, clinical officers). Conversely, policies that were more complex, such as those that include a greater number of steps, require particular skill in delivery, or involve collaboration among a number of actors, were less well implemented. Support services for PLHIV on ART, for example, exhibited minimal implementation coverage for this reason:

The support groups, it’s good in theory. [The AIDS Support Organization] has had some success… but it’s hard. A support group needs to be a group of local people who can help each other out… It’s not something that’s easy for a clinic to put in place. [R2]

### Process

All respondents identified the importance of planning for policy implementation, seen to be particularly effective in the more recent successful Option B+ roll-out. When there were small (and non-complex) amendments to policy, information was shared from the Ministry to the District Health Office and then onto facilities, usually in the form of a written memo. When there were larger changes to policy, facilities were supported through training and on-the-job mentorship. All communication was described positively. Incremental and prioritised scale-up of policies was highlighted as facilitating implementation (e.g. by targeting central regions logistically easier to access). Phased approaches enabled piloting and learning from experience to allow revision of practices for scale-up:

Option B+, it was very successful because it was planned, it was a phased approach. We did not roll out to the whole country at once. We first went for the central region then we kept on rolling-out to other regions. [R5]

The process of monitoring progress, reflecting on results and adapting was also cited as critical, as well as demand-generation activities to engage PLHIV. It was acknowledged, however, that phased scale-up was necessary due to budget and resource constraints and one respondent described the prioritisation process as “political” and “very bureaucratic” [R5], thus causing unnecessary delays in implementation.

Innovations to simplify the treatment pathway for PLHIV were cited as an explanation for why some sites in Rakai did not widely implement policies that may act as a barrier to treatment initiation, e.g. only 50% of sites implemented the policy that recommends two adherence counselling sessions prior to ART initiation.

### Context

Respondents considered constraints on complete policy implementation to be attributable to a range of contextual factors, with one respondent commenting: “infrastructure and human resources are insufficient to implement the policies” [R1]. Some respondents, however, noted that Ugandan facilities fare well in terms of HIV service delivery compared with other countries in the region and that these HDSS facilities may also perform much better than others in Uganda: “if you went to the North of the Uganda, it’s absolutely luxury down in Rakai”. [R2]

However, all underscored the importance of structural factors in inhibiting policy implementation. Human resource constraints presented a critical operational challenge for HIV services, with too few staff available to provide necessary services, primarily because of MoH funding constraints. High patient load was seen to impact on quality of care, likely in turn to inhibit patient engagement with care:

Imagine, if you will, on a Monday morning where we have a clinic officer and he has about 100 to 120 clients that are coming in. This is extremely overwhelming and therefore impacting on the quality of services. [R7]

Staff remuneration and career development prospects were also noted as problematic. Staff mobility was also emphasised, in particular, due to the loss of institutional knowledge. These issues are further perpetuated by training deficiencies, low salaries and heavy workloads. Human resource constraints were also cited for their impact on commodity management:

Sites don’t have dedicated logistics managers. The staff, she’s the doctor, she’s the nurse, she’s the soul, she’s everything. You need dedicated logistics managers. [R1]

The importance of adequate health management information systems was also commonly highlighted, with the need for adequate records to report patient history and trace patients between clinics. It was noted that there was no national or even regional tracking system. One respondent commented that improving these systems might impact on patient retention. This is of importance as more patients may be initiated on treatment earlier; retention rates will need to be improved:

Strategies for improving retention, of course one is infrastructure. If you improve infrastructure you improve clients flow, minimize waiting time, avoid stock-outs of essential drugs. I think all those we need to do … then tracking those clients who didn’t get lost to follow-up. [R1]

The political context was also influential, notably on policy and implementation gaps for testing and treatment services for MARPs, and it was noted that facilities may have been fearful to openly report provision of these services: “Because they won’t report they are doing that as it is illegal. There will be a lot of places that will be providing services for those people but not in name”. [R2]

## Discussion

This study has reviewed the implementation of HIV policy in Uganda within two rural HDSS in 2013. To our knowledge, this is the first comprehensive assessment conducted in the region that specifically contrasts the policy and practice of a range of indicators determinant in ensuring progression of PLHIV from diagnosis through to retention on long-term treatment.

The findings show that the majority of policies that may influence access to HCT, access to treatment for those diagnosed positive or retention in care were widely implemented across facilities in both sites. This is a notable achievement given the inherent health system weaknesses that can obstruct effective service delivery, which has been previously observed in many settings in Uganda [5].

There were, however, shortcomings either in both sites or in several areas. There were more commonalities than differences in implementation between the sites, with a similar pattern of practice observed for each policy (i.e. if Kyamulibwa reported wide implementation of a policy, so did Rakai), which suggests that factors influencing implementation may be common to both sites. However, Rakai achieved higher levels of implementation for policies relating to provision of treatment. Both Kyamulibwa and Rakai reported mixed implementation of policy indicators influencing retention in care (notably indicators providing support for PLHIV).

The qualitative findings identified a range of influences on policy implementation, usually facilitating practice rather than inhibiting. The commonalities between the implementation analysis and reports from informants give confidence to the accuracy of the self-report in the facility survey. Respondents highlighted several positive aspects of the policy cycle in Uganda, including substantial donor investment and support, phased implementation and routine hierarchical processes for information dissemination. However, informants strongly emphasised the inadequate quality of care and gaps in service provision considered to be common in the Ugandan context [5].

The pattern of implementation appears to follow the strength of recommendation and evidence-based for formulating it. Policies providing impactful results in the short term also appeared to be a strong enabling factor in implementation, as noted with the successful roll-out of Option B+. This may indicate evidence-driven policy implementation or reflect the donor-driven political environment in which it is preferable to deliver tangible results and maximum impact with the financial support they provide [5, 14]. As noted by our informants, such approaches may not always align with national priorities or local needs, with prevention activities often under-resourced. Emphasis by donors in support of medical management of HIV through ART may result in oversight of implementation of interventions supporting patient coordination or retention in care, which strengthen the comprehensive care package necessary for PLHIV to be maintained in the long-term care system. Retention strategies tend to have variable efficacy or may be complex to implement (as with patient support groups, noted by key informants) [15]. This leads to a “vicious cycle” in which donors have little incentive to support such strategies, and less investment will result in less impact and less future evidence. The comparison is particularly stark when reviewed alongside implementation of clinical policies such as Option B+, as the successes will be harder to estimate or will appear over longer time horizons.

These oversights are particularly crucial given the recent changes to WHO guidance to recommend immediate ART initiation following HIV. The change in policy from Option A to Option B+ shares some similarities with this recent change in treatment eligibility by the WHO, and efforts should be made to learn from Uganda’s response to the former. This study indicates Option B+ was widely implemented across facilities within a short time frame due to support (political and economic) from individuals and institutions involved. Further, given that the policy was a simplification of a previous protocol, the training requirements were minimal, which enabled lower cadres of staff to perform the task. This bodes well for implementation of immediate ART as this is also a simplification of previous treatment protocol. However, the capacity of HIV programmes to retain high patient numbers in care may be of concern. In this study, facilities were already suffering stock-outs and human resource challenges; these systemic weaknesses are likely to be amplified with higher patient loads and development of resistance to ART may become increasingly problematic [16, 17]. While these changes in policy have the potential to markedly improve individual and population health, significant additional financial resources will need to be mobilised in the short term for commodities, laboratory facilities and facility-level and personnel costs [18, 19]. Pre-existing gaps in policy and practice along the diagnosis-to-treatment cascade could limit the effectiveness of this strategy [4].

Funding from international donors continues to be the main route through which HIV services are financed in Uganda; in 2011, it was estimated that 95% of the programme was funded by external donors, with PEPFAR responsible for 73% [5]. Therefore, support for implementation of policies to improve the comprehensive care package provided to PLHIV will require donor backing. Key informants in this study reported on the complex dynamic of misalignment of goals between the government and donors in assessing priorities, making reference mostly to a lack of support for prevention activities, but also noting that support for health system strengthening would not be available [20]. The Uganda AIDS Commission 2013 Country Progress Report indicate there is a need for support or leadership for strategies that enable strengthening of coordination of care and care provision for the later stages of the treatment continuum in which PLHIV should ultimately spend more time [5]. The WHO’s 2015 ARV Guidelines include a number of operational recommendations on how to simplify and streamline care in order to reduce clinic burdens, and the adoption of such strategies should be considered if the programme is to sustain PLHIV in care over longer time horizons [21].

### Study limitations

The facilities may not be representative of facility performance across Uganda, as routine research activities within the HDSS may have benefited service delivery. Health facility survey data may be affected by reporting bias, resulting in an overestimation of implementation, as managers may have been inclined to report practice in the best light. However, the number of gaps in implementation reported and the alignment with findings from key informants gives us confidence that reporting was relatively truthful. The policy and practice comparison presents a picture of a well-functioning health system; however, the qualitative interviews provide an alternative view. Human resources, infrastructure and health management information system limitations may be limiting the quality of care that is provided to patients, which might influence patient engagement with the health system [15]. This generates hypotheses for further investigation to develop a deeper understanding of policy implementation. ALPHA’s ongoing qualitative research on the HIV continuum of care among people living with HIV in the HDSS may further inform findings on health facility performance and provider behaviour in this setting and help validate our findings. “Mystery client” patient observations, in particular, would prove insightful. This analysis was also not able to estimate the health impact of each policy, so it is difficult to draw conclusions about the effect that implementation gaps have on epidemiological outcomes.

## Conclusions

This study provides insight into implementation status of MoH policies for HIV care in two HDSS sites in Uganda in 2013. While overall implementation was strong across the majority of policies, implementation coverage was frequently lower than 100% for many policies implying there is still a gap in complete implementation of most national HIV policies in Uganda, even in the relatively well-resourced HDSS. Key informants indicated that quality of care provided may have been limited in this setting, however, and noted the emphasis from donors to fund treatment, with minimal support for policies relating to coordination and retention of patients. Attention should be given to closing gaps in implementation (both at stages of the cascade and in the variability of facilities to adopt national policies) to improve the comprehensive care package that should be provided along the diagnosis-to-treatment cascade. This is particularly important as the country endeavours to respond to changing global guidance from the WHO, notably the change to immediate ART eligibility. While focus on entry into the care cascade remains vitally important, failure to ensure maintenance in care downstream in the care pathway could result in greater patient attrition from already overburdened facilities. Novel service delivery strategies to improve efficiency should be considered.

## Abbreviations

ALPHA: Network for Analysing Longitudinal Population-based data on HIV in Africa
ART: Antiretroviral treatment
CSW: Commercial sex workers
CTX: Co-trimoxazole
HCT: HIV counselling and testing
HDSS: Health and demographic surveillance site
HIV: Human immunodeficiency virus
HMIS: Health management information systems
HR: Human resources
IDU: Injection drug user
IPT: Isoniazid Preventive Therapy
KI: Key informant
MARP: Most-at-risk persons
MoH: Ministry of Health
MSM: Men who have sex with men
NGO: Non-governmental organisation
OI: Opportunistic infection
PEPFAR: Presidents Emergency Fund for AIDS Relief
PMTCT: Prevention of mother to child transmission
TB: Tuberculosis
UNAIDS: Joint United Nations Department for HIV/AIDS
WHO: World Health Organization
